# Stabilometric Correlates of Motor and Motor Imagery Expertise

**DOI:** 10.3389/fnhum.2021.741709

**Published:** 2022-01-13

**Authors:** Franck Di Rienzo, Pierric Joassy, Thiago Ferreira Dias Kanthack, François Moncel, Quentin Mercier, Christian Collet, Aymeric Guillot

**Affiliations:** ^1^Univ Lyon, Université Claude Bernard Lyon 1, Laboratoire Interuniversitaire de Biologie de la Motricité, Villeurbanne Cedex, France; ^2^Institut Universitaire de France, Paris, France

**Keywords:** cognition, posture, balance, performance, mental imagery

## Abstract

Motor Imagery (MI) reproduces cognitive operations associated with the actual motor preparation and execution. Postural recordings during MI reflect somatic motor commands targeting peripheral effectors involved in balance control. However, how these relate to the actual motor expertise and may vary along with the MI modality remains debated. In the present experiment, two groups of expert and non-expert gymnasts underwent stabilometric assessments while performing physically and mentally a balance skill. We implemented psychometric measures of MI ability, while stabilometric variables were calculated from the center of pressure (COP) oscillations. Psychometric evaluations revealed greater MI ability in experts, specifically for the visual modality. Experts exhibited reduced surface COP oscillations in the antero-posterior axis compared to non-experts during the balance skill (14.90%, 95% CI 34.48–4.68, *p* < 0.05). Experts further exhibited reduced length of COP displacement in the antero-posterior axis and as a function of the displacement area during visual and kinesthetic MI compared to the control condition (20.51%, 95% CI 0.99–40.03 and 21.85%, 95% CI 2.33–41.37, respectively, both *p* < 0.05). Predictive relationships were found between the stabilometric correlates of visual MI and physical practice of the balance skill, as well as between the stabilometric correlates of kinesthetic MI and the training experience in experts. Present results provide original stabilometric insights into the relationships between MI and expertise level. While data support the incomplete inhibition of postural commands during MI, whether postural responses during MI of various modalities mirror the level of motor expertise remains unclear.

## Introduction

Motor imagery (MI) is the mental representation of a movement without any overt execution (Jeannerod, 1994). Psychometric, behavioral, and neurophysiological similarities between MI and physical practice of the same action have been the focus of a large number of scientific investigations over the last two decades (Guillot and Collet, 2005; Collet et al., 2011). Functional brain imaging experiments provided compelling evidence in support of a functional equivalence hypothesis between MI and physical practice of the same task (Lotze and Halsband, 2006; Munzert and Zentgraf, 2009; Hétu et al., 2013). The cerebral networks recruited during MI largely overlap those recruited during the physical practice of the same action (e.g., Gerardin et al., 2000; Ehrsson et al., 2003; Hanakawa et al., 2008), including cortical structures such as premotor and primary motor cortices, as well as subcortical regions such as the basal ganglia and the cerebellum (Lotze et al., 1999; Gerardin et al., 2000; Hardwick et al., 2018). There is thus a consensus that MI elevates the cognitive demand on cerebral motor networks and leverages experience-based plasticity (Feltz and Landers, 1983; Driskell et al., 1994; Jackson et al., 2001; Di Rienzo et al., 2016). MI modalities refer to the sensory information focused during the mental representation. Visual MI classically involves first (performing the movement oneself) or third (as an external observer) person perspectives, whereas kinesthetic imagery focuses on proprioceptive information, e.g., muscle contractions, vestibular information, and balance (White and Hardy, 1995). Visual and kinesthetic modalities involve partially distinct cerebral networks, with a more consistent involvement of sensorimotor and parietal structures during kinesthetic MI, and a more pronounced involvement of occipital cortical areas during visual MI (Guillot et al., 2009).

At the peripheral level, there is scientific evidence that both autonomic and somatic motor command signals are produced during MI and replicate neurophysiological responses inherent to the actual motor preparation (Collet and Guillot, 2009; Collet et al., 2011; Guillot et al., 2012). In other words, MI reproduces with reduced magnitude the neurophysiological markers of readiness states associated with the actual motor preparation, e.g., heart rate, ventilatory rate and skin conductance increases as corollary of anticipated increases of energy expenditure (for a more exhaustive development, see Collet et al., 2013). A debated issue relates to the recruitment of somatic effectors during MI (Wehner et al., 1984; Bonnet et al., 1997; Guillot et al., 2007; Lebon et al., 2008). The simulation theory postulates low-threshold muscle activation without contraction during MI (Gandevia et al., 1997; Nikulin et al., 2008). Several electromyography experiments reported subliminal yet task-specific muscle activation patterns (for pioneer insights, see Jacobson, 1930, 1932). Other experiments refuted this hypothesis, with a possible account of the MI content and the purpose of the MI intervention (Dickstein et al., 2005; Kanthack et al., 2017). Whether peripheral responses during MI originate from an incomplete inhibition of preparatory motor commands by suppressive mechanisms or result from an incomplete facilitation of efferent pathways, hence without motor inhibition *per se*, remains unresolved (for reviews, see Stinear, 2010; Guillot et al., 2012).

Motor preparation involves cognitive operations associating action goals with motor commands (i.e., inverse model) and motor commands with their sensory consequences (i.e., forward model; Wolpert et al., 2011). It has long been established that voluntary movements involve postural reactions designed to cancel their disturbing effects on balance (Bouisset and Zattara, 1981, 1990; Massion, 1992). Anticipatory postural adjustments refer to postural regulations preceding the movement (Massion, 1992). Indeed, their onset occurs before, or perfectly time-locked, to the activation of agonists, as shown for upper limb/trunk movements in humans (Lee, 1980; Cordo and Nashner, 1982; Crenna et al., 1987). In other words, mechanical perturbations of the body posture associated with voluntary movements can be counteracted by the central nervous system in a feedforward manner (Bouisset and Zattara, 1990; Massion, 1992; Aruin et al., 1998; Klous et al., 2011). This is a fundamentally distinct mechanism from the tonic regulation of the body posture based on spinal reflexes. Interestingly, MI is hypothesized to reproduce forward and inverse modeling operations inherent to the feedforward modes of action control (see Wolpert and Ghahramani, 2000; Grush, 2004). Postural commands indicative of anticipatory postural adjustments should thus be detectable during MI.

Rodrigues et al. (2003) demonstrated that, compared to mental calculation, visual imagery of plantar flexions was not associated with increased Centre of Pressure (COP) sway, i.e., the vertical projection of the center of mass on the ground where the vector sum of ground-reaction forces are applied in an inverted pendulum model of human balance control. Conversely, kinesthetic MI increased COP oscillations (Rodrigues et al., 2003, 2010). Motor predictions associated with kinesthetic, but not visual MI, appeared to generate residual postural commands (see also Stins et al., 2015). These results were replicated by Grangeon et al. (2011), who investigated the stabilometric correlates of visual and kinesthetic MI of counter-movement jumps. Although decreased postural sway was recorded during MI compared to control conditions (i.e., absence of motion and mental calculation), postural sway variability was higher during MI of the counter movement jump compared to MI of finger movements (see Lemos et al., 2014 for analogous findings). Boulton and Mitra (2015) recently addressed the hypothesis that central inhibition, whenever present during MI, would be largely blinded to postural motor commands processing since these are primarily processed subcortically (Jordan et al., 1992; Lalonde and Strazielle, 2007; Yin, 2017). They recorded reduced postural sway as participants imagined arm movement in the direction of the body stance. Noteworthy, these authors controlled for a potential peripheral origin of the postural regulations by requiring participants to imagine an additional load attached to their forearm, hence ensuring that the postural commands were not due to the central integration of proprioceptive afferents during forward modeling operations. It seems overall that postural motor commands detected during MI reflect distinct degrees of embodiment according to the MI modality, and reflect physical demands in terms of balance control (for a pioneering discussion, see Guillot and Collet, 2005; Collet and Guillot, 2009).

Improved balance control after MI training was associated with the capacity to engage the central nervous system into the processing of motor command signals targeting the somatic effectors responsible for balance control (Fansler et al., 1985; Hamel and Lajoie, 2005; Taube et al., 2014). Experience-based plasticity associated with the motor expertise is mirrored in the cerebral activations patterns during MI (for reviews, see Olsson and Nyberg, 2010; Debarnot et al., 2014; Di Rienzo et al., 2016; Mizuguchi and Kanosue, 2017). A more focused and intense recruitment of brain motor system regions was found in experts (Lotze et al., 2003; Milton et al., 2007, 2008). Ross et al. (2003) reported a negative relationship between the activation of the supplementary motor area and the cerebellum during MI of a golf swing and the golf handicap. The authors argued for a more efficient management of mental resources during motor representations in experts. By contrast, novices may experience higher mental strain, specifically when engaging in MI from a kinesthetic perspective, which requires more experience of the task (Hardy and Callow, 1999; Féry, 2003; Guillot et al., 2004; Girón et al., 2012). For this reason, visual MI is considered relevant to promote early motor learning (Guillot et al., 2004), while kinesthetic MI might more extensively improve performance in experts (Hardy and Callow, 1999). Guidelines for efficient use of MI modalities in training interventions thus appear to follow a hierarchical model. Visual MI may be privileged during the first stages of learning, whereas kinesthetic MI—considered more difficult than visual MI—may be administered in experts with greater experience of the movement. This postulate is supported by recent functional brain imaging findings indicating that non-expert participants spontaneously engaged in visual MI strategies when requested to perform kinesthetic MI of difficult movements (Mizuguchi et al., 2016). However, disentangling whether the postural correlates of MI are influenced by the level of motor expertise, and whether such effects may vary according to the MI modality, remains unanswered. This question is particularly relevant in the current context of growing interventions with MI training to improve balance control in sports and rehabilitation (Hamel and Lajoie, 2005; Nagar and Noohu, 2014; Taube et al., 2014; Abraham et al., 2016; Saruco et al., 2017).

In the present study, we investigated the postural correlates of visual and kinesthetic MI in experts and non-experts of a balance skill. We hypothesized that kinesthetic MI, but not visual MI, could result in reduced postural sway considering the more embodied nature of this modality (Jeannerod, 1995). We also hypothesized greater embodiment of the postural correlates of balance skills during MI in experts compared to non-experts. Experts have greater experience of the task due to years of training practice, yielding experience-based cerebral plasticity (Di Rienzo et al., 2016). They may have a greater capacity to build motor predictions from procedural memories—particularly during kinesthetic MI (Guillot et al., 2008). This hypothesis is in keeping with Paillard (2017) statements that sporting expertise is associated with a greater capacity to integrate and use proprioceptive information to implement efficient balance control strategies (e.g., Noé and Paillard, 2005; Paillard and Noé, 2006; Paillard et al., 2011).

## Material and Method

### Participants

Twenty-eight subjects (14 women, 21 right-handed) volunteered to participate in the experiment. A first group (EXPERTS) included gymnasts of regional to national levels (*n* = 13, 167.61 ± 8.35 cm, 64.46 ± 10.15 kg, 20.15 ± 3.62 years). EXPERTS underwent a minimum of 3 h of gymnastics training per week over the last 2 years preceding the experiment (9.30 ± 4.31 years of practice). A second group (NON-EXPERTS) included non-gymnasts without any specific experience of balance tasks (*n* = 15, 170.87 ± 8.74 cm; 66.13 ± 14.88 kg; 24.87 ± 2.59 years). EXPERTS and NON-EXPERTS had no medical history of locomotor injuries and cognitive impairments which could have confounded the results, e.g., postural control impairments. No information regarding the aims and scopes of the study was provided until the completion of the design. The present experiment was approved by the local ethics committee (IRB 2019-A01732-55). All participants provided a written informed consent form according to the statements of the Declaration of Helsinki (2013^1^). Parental authorization was obtained for minor participants.

### Experimental Design

The experimental design consisted of (i) a baseline evaluation of postural performance from the stance and (ii) an evaluation of postural performance under four experimental conditions administered in a random order (block randomization).

**Figure 1:**
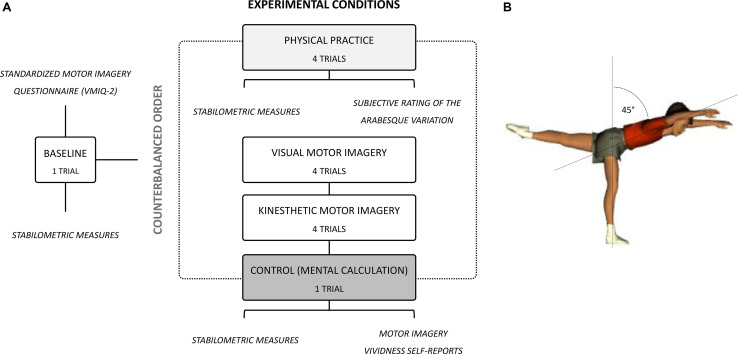
**(A)** Flowchart of the experimental design. **(B)** Graphic representation of the arabesque variation administered during stabilometric recordings under physical practice, visual MI, kinesthetic MI, and control experimental conditions.

Baseline and experimental condition evaluations were carried on using a stabilometric platform (WIN-POSTURO, Médicapteurs, France). Participants starred at a cross mark placed against the wall, 5 m ahead of them. For baseline evaluations, we recorded stabilometric indexes as participants remained motionless in a stand-up position for 15 s, both feet in contact with the postural platform, arms alongside the body (BASELINE, 1 trial). Then, we randomly administered four experimental conditions. During a first condition, we measured postural performance as participants performed a variation of the “arabesque” gymnastics skill. While standing on their dominant leg on the stabilometric platform, they leaned forward while constantly keeping their non-dominant hip, knee and ankle extended, to reach 45° of body inclination. They maintained this posture for 15 s (ACTUAL PRACTICE, 4 trials; Figure 1A). Participants verbally indicated the onset of the trial to the experimenters after having stabilized the arabesque body posture. During second and third conditions, participants remained motionless in a stand-up position identical to the BASELINE, but respectively performed 15 s of visual MI (VMI, four trials) or kinesthetic MI (KMI, four trials) of the arabesque. They verbally indicated the onset of each trial to the experimenter. During the last condition, participants remained motionless in a stand-up position identical to the BASELINE for 15 s, but performed a mental calculation control task (CONTROL, 1 trial). Starting from 1, they added the immediately following number to the result obtained and iterated this operation (i.e., 1, 3, 7, 15, etc.). This controlled the state of attentional focus during VMI and KMI. Eyes remained open during all experimental conditions to prevent any variation in postural sway that would not be related to cognitive and/or motor processes.

### Dependent Variables

#### Stabilometric Measures

The stabilometric platform enabled online recordings of vertical ground reaction forces. Data acquisition (40 Hz) was handled by the Win-Posturo^©^ software (Balma, France). The COP was determined as the weighted average of vertical ground reaction forces. We first collected the path length of COP oscillations in the medial-lateral and anterior-posterior axes (ML-Length and AP-Length, respectively). We also collected the area of the ellipse encompassing 90% of the COP coordinates throughout the trial duration (Surface). We finally measured the ratio between the total length of COP oscillations and their surface of displacement (Length-by-Surface). This variable conveys a global index of energy expenditure (Gagey and Weber, 1999). Length-by-Surface ratios should be close to one. An increase in length-by-surface indicated an increase in energy expenditure to maintain balance. Conversely, a decrease in length-by-surface reflected energy sparing through efficient postural control strategies.

The median value of the four trials of each experimental condition was normalized with reference to the median BASELINE value based on the following formulae:


$$Stabilometric\;data_{\left(normalized\right)}=\frac{Stabilometric\;data_{\left(Actual\;practice,\;VMI,\;KMI,\;CONTROL\right)}}{Stabilometric\;data_{\left(Baseline\right)}}*100$$

#### Subjective Variables

##### Subjective Rating of the Arabesque Performance

During ACTUAL PRACTICE, trials were assessed from a subjective 5-point scale ranging from 0 (“fall or failed execution”) to 4 (“perfect technical execution of the arabesque variation throughout the trial duration”) by a gymnastics judge of national-level blinded to the purpose of the study and participants’ assignment to experimental groups.

##### Subjective Evaluation of MI Ability

We administered the revised Vividness of Movement Imagery Questionnaire (VMIQ-2 Roberts et al., 2008) before conducting stabilometric measures (Figure 1). The VMIQ-2 provides a global index of MI ability. The VMIQ-2 specifically measures the difficulty to perform internal visual MI (IVI), external visual MI (EVI), and kinesthetic MI (KMI) across 12 items. For each MI modality, each item is evaluated on a 5-point Likert-scale ranging from 1 (“very clear and vivid mental representation”) to 5 (“no mental representation”). The VMIQ-2 demonstrated adequate internal reliability with Cronbach’s alphas > 0.90 for all dimensions.

After each VMI and KMI trial, we collected subjective ratings of the perceived MI vividness from a Likert-type scale ranging from 1 (“Absence of visual/proprioceptive perception of the movement”) to 6 (“Same visual/proprioceptive perception of the movement as during physical practice”).

### Statistical Analysis

Due to deviations from normality (Q-Q plots), we used R (R Core Team, 2018) and the package ARTool (Kay and Wobbrock, 2019) to perform a nonparametric factorial analysis of stabilometric data (Wobbrock et al., 2011). The procedure consists of a preliminary step of data alignment based on the mean estimates of main and interaction effects of a factorial model, followed by rank assignment (for further details, see Wobbrock et al., 2011). We applied the Aligned Rank Transform (ART) procedure to a series of linear mixed effects models with by-subjects random intercepts. We first analyzed data collected during the baseline. For each stabilometric variable (ML-length, AP-length, Surface, Length-by-surface), we entered the fixed effect of GROUP (EXPERTS, NON-EXPERTS). Stabilometric measures during physical practice of the arabesque variation were analyzed using a similar model. Stabilometric data collected during MI conditions were analyzed separately, by running a series of linear mixed effects models testing the effect of CONDITION (VMI, KMI, CONTROL) and GROUP (EXPERTS, NON-EXPERTS), with interaction terms. For psychometric variables (VMIQ scores and subjective ratings of the arabesque performance by the judge), we only entered the fixed effect of GROUP (EXPERTS, NON-EXPERTS) in the random-coefficient regression model. To investigate a link between motor and MI expertise, we calculated, for each variable obtained from stabilometric measures during physical practice, random-coefficient regression models with the corresponding variable recorded during VMI and KMI, as well as vividness self-reports during VMI and KMI as regressors. Also, the relationship between stabilometric variables recorded during ACTUAL PRACTICE, VMI, and KMI and the years of training practice in experts was tested using linear regression models. Visual inspection of the residual plots did not reveal deviations from homoscedasticity or normality. The statistical significance threshold was set for a type 1 error rate of 5%. Partial eta-squared ($\eta_{p}^{2}$) were reported as measure of effect size. For *post hoc* investigations, we used *emmeans*^2^ to calculate planned contrasts of estimated marginal means (least-squares means) from the factors and factors’ combination of the linear models. We applied Holm’s sequential corrections to control the false discovery rate (Holm, 1979).

## Results

### Stabilometric Analysis

GROUP did not influence the raw baseline values for all stabilometric variables collected from during BASELINE (all *p* > 0.05; Table 1).

**Table 1:** Raw data before normalization for COP variables recorded in the time domain.

	Surface (mm^2^)				
	Control	KMI	VMI	Actual practice	Baseline
Non-Experts	77.46 ± 76.36	128.9 ± 72.34	91.12 ± 50.04	2,316.05 ± 913.41	121.13 ± 99.56
Experts	92.42 ± 64.73	213.11 ± 248.71	103.9 ± 61.62	2,451.63 ± 1118.21	143 ± 73.29
	**ML-Length (mm)**				
	**Control**	**KMI**	**VMI**	**Actual practice**	**Baseline**
Non-Experts	73.24 ± 37.37	80.78 ± 30.47	70.89 ± 29.7	656.33 ± 136.36	92.15 ± 34.05
Experts	91.26 ± 26.07	92.45 ± 28.86	78.65 ± 15.17	624.05 ± 143.79	92.07 ± 23.91
	**AP-Length (mm)**				
	**Control**	**KMI**	**VMI**	**Actual practice**	**Baseline**
Non-Experts	86.28 ± 39	92.53 ± 28.88	83.6 ± 21.43	692.06 ± 188.46	94.09 ± 25.8
Experts	108.52 ± 29.47	105.54 ± 28.7	93.08 ± 22.59	646.37 ± 169.26	102.45 ± 17.98
	**Length-by-surface (mm^−1^)**				
	**Control**	**KMI**	**VMI**	**Actual practice**	**Baseline**
Non-Experts	0.3 ± 0.1	0.3 ± 0.08	0.28 ± 0.08	0.51 ± 0.32	0.34 ± 0.09
Experts	0.37 ± 0.06	0.33 ± 0.05	0.31 ± 0.05	0.48 ± 0.29	0.34 ± 0.04

*COP, center of pressure; KMI, Kinesthetic MI; VMI, visual MI*.

#### Analysis of the Arabesque Performance

**Figure 2:**
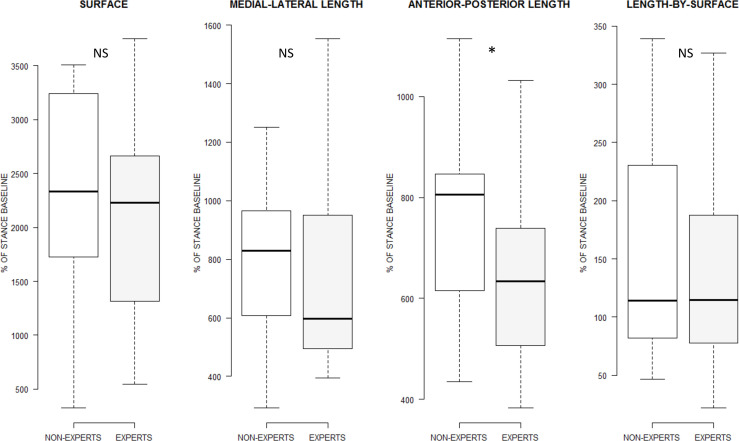
Boxplot display of the main effect of GROUP on the variables recorded from stabilometric measures for the physical practice condition. Only the length of COP oscillations in the anterior-posterior axis differed between experts and non-experts. *p < 0.05, NS: Not statistically significant.

The linear mixed effects analysis with ART revealed that the main effect of GROUP did not affect the Surface of COP oscillations (*F*_(1, 26)_ = 0.43, *p* = 0.51, $\eta_{p}^{2}$ = 0.01), ML-length (*F*_(1, 26)_ = 0.56, *p* = 0.46, $\eta_{p}^{2}$ = 0.02), and Length-by-surface (*F*_(1, 26)_ = 0.11, *p* = 0.73, $\eta_{p}^{2}$ = 0.00). However, GROUP affected AP-length (*F*_(1, 26)_ = 3.23, *p* = 0.04, $\eta_{p}^{2}$ = 0.11), with reduced AP-length in EXPERTS compared to NON-EXPERTS (*p*_(k = 1)_ = 0.04; Figure 2).

#### Analysis of the Stabilometric Data Recorded During Mental Practice Conditions

**Figure 3:**
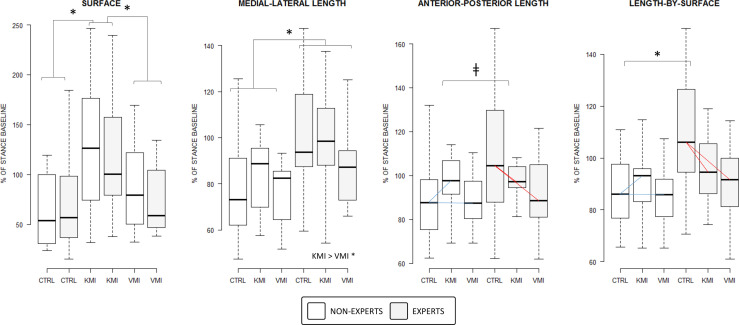
Boxplot representation of the CONDITION by GROUP interaction effect on stabilometric variables calculated in the time domain. Differences of differences are represented by a color code emphasizing distinct slopes patterns materializing differences across experimental conditions in the experts groups and non-experts for the length of COP displacement in the anterior-posterior axis and length-by-surface of COP displacement only. *p < 0.05, ^†^*p* < 0.10. CTRL: Control. KMI: Kinesthetic Motor Imagery. VMI: Visual Motor Imagery.

The CONDITION × GROUP interaction affected the AP-Length (*F*_(2, 52)_ = 2.38, *p* = 0.04, $\eta_{p}^{2}$ = 0.09) and approached the statistical significance threshold for Length-by-surface (*F*_(2, 52)_ = 3.03, *p* = 0.05, $\eta_{p}^{2}$ = 0.10), but did not affect ML-Length (*F*_(2, 52)_ = 1.64, *p* = 0.20, $\eta_{p}^{2}$ = 0.06) and the Surface of COP oscillations (*F*_(2, 52)_ = 0.22, *p* = 0.79, $\eta_{p}^{2}$ = 0.00). The Length-by-surface decrease pattern observed in EXPERTS between CONTROL and VMI was absent in NON-EXPERTS (*p*_(k = 3)_ = 0.04). Also, the Length-by-surface decrease recorded in EXPERTS between CONTROL and KMI was not present in NON-EXPERTS (*p*_(k = 3)_ = 0.04; Figure 3). Likewise, the decrease pattern observed for AP-length in EXPERTS between CONTROL and VMI as well as between CONTROL and KMI was marginally distinct from the pattern in NON-EXPERTS (both *p*_(k = 3)_ = 0.09). Noteworthy, stabilometric values during the CONTROL condition were similar between EXPERTS and NON-EXPERTS for both Length-by-surface (*p*_(k = 3)_ = 0.19) and AP-length (*p*_(k = 3)_ = 0.33). By contrast, ML-length and Surface of COP oscillations exhibited comparable patterns across conditions in the two groups (Figure 3).

The main effect of CONDITION affected the Surface (*F*_(2, 52)_ = 10.65, *p* < 0.001, $\eta_{p}^{2}$ = 0.29), ML-Length (*F*_(2, 52)_ = 5.05, *p* = 0.009, $\eta_{p}^{2}$ = 0.16), and the Length-by surface (*F*_(2, 52)_ = 4.31, *p* = 0.02, $\eta_{p}^{2}$ = 0.14) but only approached the statistical threshold for AP-Length (*F*_(2, 52)_ = 2.79, *p* = 0.07, $\eta_{p}^{2}$ = 0.10). The surface of COP oscillations during KMI was higher compared to that recorded during CONTROL (*p*_(k = 3)_ < 0.001) and VMI (*p*_(k = 3)_ = 0.01), while there was no difference between VMI and CONTROL (*p*_(k = 3)_ = 0.13; Figure 3). Also, ML-Length during KMI was greater compared to VMI (*p*_(k = 3)_ = 0.007), but there was no difference between VMI and CONTROL, or between KMI and CONTROL (*p*_(k = 3)_ = 0.17 and *p*_(k = 3)_ = 0.15, respectively). The linear mixed effects analysis with ART eventually revealed that the main GROUP effect affected ML-Length (*F*_(1, 26)_ = 5.28, *p* = 0.03, $\eta_{p}^{2}$ = 0.17) and the Length-by-surface (*F*_(1, 26)_ = 4.81, *p* = 0.04, $\eta_{p}^{2}$ = 0.16), but did not affect AP-Length (*F*_(1, 26)_ = 1.40, *p* = 0.24, $\eta_{p}^{2}$ = 0.05) and Surface (*F*_(1, 26)_ = 0.00, *p* = 0.97, $\eta_{p}^{2}$ = 0.00) of COP oscillations. ML-Length in the EXPERT group was higher compared to that in the NON-EXPERT group (*p*_(k = 1)_ = 0.03).

### Analysis of Subjective Measures

#### Subjective Ratings of Performance

**Figure 4:**
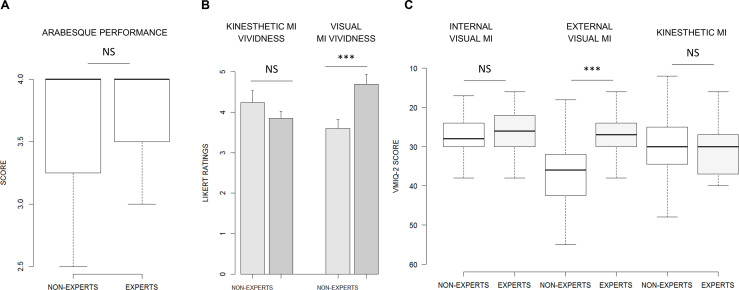
**(A)** Boxplot summarizing the GROUP effect on subjective ratings of the arabesque performance. **(B)** Barplot display of the GROUP effect on kinesthetic and visual MI vividness self-reports. **(C)** Summary of the GROUP effect on VMIQ-2 dimensions. ***p < 0.001. MI: Motor Imagery.

There was no effect of GROUP on the subjective ratings of the arabesque performance (*F*_(1, 26)_ = 0.22, *p* = 0.64, $\eta_{p}^{2}$ = 0.01; Figure 4A).

#### Subjective Ratings of MI Ability

GROUP did not affect MI ease/difficulty ratings during KMI (*F*_(1, 26)_ = 1.05, *p* = 0.30, $\eta_{p}^{2}$ = 0.04). However, MI vividness ratings during VMI were affected by GROUP (*F*_(1, 26)_ = 14.53, *p* < 0.001, $\eta_{p}^{2}$ = 0.35). MI vividness scores were higher in EXPERTS (4.96 ± 0.85) compared to NON-EXPERTS (3.60 ± 0.66; 1.09 ± 0.28, *p* < 0.001; Figure 4B).

GROUP affected VMIQ-2 scores for the EVI dimension (*F*_(1, 26)_ = 10.71, *p* = 0.003, $\eta_{p}^{2}$ = 0.28). EVI scores were higher in the NON-EXPERTS (36.93 ± 10.03) compared to EXPERTS (26.62 ± 5.66; *p* < 0.001). Conversely, we found no group effect for IVI (26.35 ± 6.05) and KMI (29.71 ± 9.17) dimensions of the VMIQ-2 (*F*_(1, 26)_ = 0.43, *p* = 0.51, $\eta_{p}^{2}$ = 0.02; *F*_(1, 26)_ = 0.00, *p* = 0.97, $\eta_{p}^{2}$ = 0.00, respectively; Figure 4C).

### Correlation Analyses

**Figure 5:**
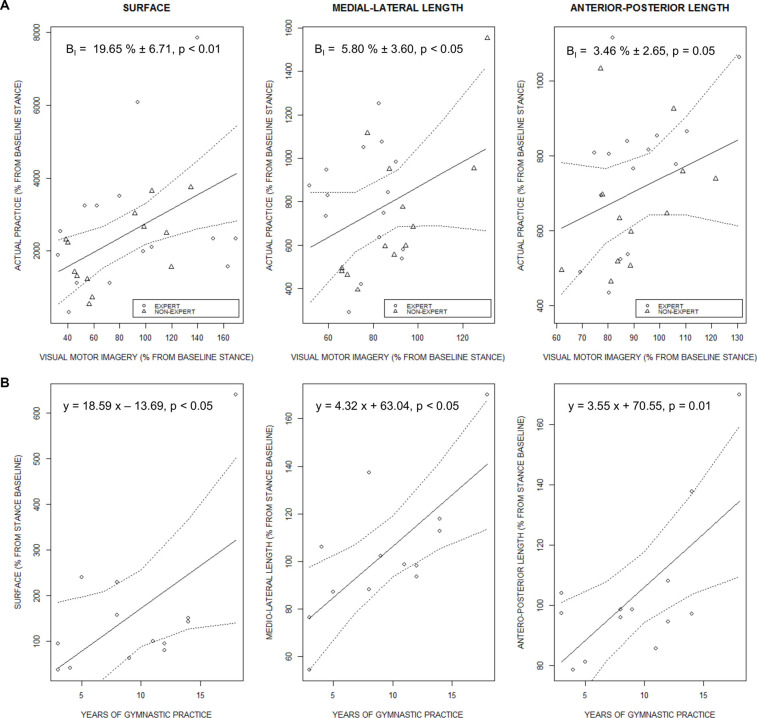
**(A)** Fitted estimates of the regression analysis carried on variables extracted from stabilometric measures during actual practice of the arabesque variation, using stabilometric measures during VMI as regressor. The regression slope is presented with 95% confidence intervals (dotted lines). **(B)** Regression slope of the linear model carried on stabilometric variables recorded during KMI and the amount of training practice in experts.

The surface of COP oscillations during ACTUAL PRACTICE was predicted by the surface of COP oscillations during VMI (*F*_(1, 23)_ = 5.50, *p* = 0.01, $\eta_{p}^{2}$ = 0.19). Comparable predictive relationships emerged for ML-Length (*F*_(1, 23)_ = 3.99, *p* = 0.02, $\eta_{p}^{2}$ = 0.15) and AP-Length (*F*_(1, 23)_ = 3.24, *p* = 0.04, $\eta_{p}^{2}$ = 0.12; Figure 5A). Stabilometric data recorded during KMI, however, did not predict any of the stabilometric measures during ACTUAL PRACTICE of the arabesque variation. Interestingly, vividness self-reports collected for KMI predicted both the Surface (*F*_(1, 26)_ = 0.43, *p* = 0.003, $\eta_{p}^{2}$ = 0.02) and Length-by-surface (*F*_(1, 26)_ = 0.43, *p* = 0.03, $\eta_{p}^{2}$ = 0.02) of COP sway measured during ACTUAL PRACTICE, whereas there was no such relationship for self-reports of VMI vividness.

Regression analyses carried on the stabilometric indexes of KMI and the years of training practice in EXPERTS revealed a positive relationship for the Surface (*F*_(1, 11)_ = 4.93, *p* = 0.04, *R*-squared = 0.31), ML-Length (*F*_(1, 11)_ = 11.63, *p* = 0.05, *R*-squared = 0.51) and AP-length of COP oscillations (*F*_(1, 11)_ = 9.39, *p* = 0.01, *R*-squared = 0.46; Figure 5B). However, there was no relationship between the number of years of training practice and the stabilometric variables recorded during ACTUAL PRACTICE, VMI, or between the number of years of training practice and the vividness self-reports under VMI and KMI.

## Discussion

The present study investigated the stabilometric correlates of MI in expert and non-experts of balance skills. The balance skill required fine postural control strategies to minimize the disturbing effects of changes in body posture on balance. Compared to non-experts, experts achieved a more efficient postural control during the physical performance of the balance skill, and exhibited distinct stabilometric response patterns during visual and kinesthetic MI compared to the control condition. A predictive relationship between the postural correlates of visual MI and those of the physical performance of the arabesque variation was present. In experts, the training experience positively predicted stabilometric response during kinesthetic MI, but not during visual MI. Present findings support that greater motor expertise of balance skills may be associated with distinct MI ability profiles.

Unexpectedly, the subjective performance evaluation by a blinded external evaluator revealed no difference between experts and non-experts. Subjective scorings remain the standard approach to performance evaluation in high-level competitive gymnastics events. However, the balance skill administered in the present design remains accessible even for non-gymnasts. This possibly yielded ceiling effects during the subjective assessment. Administering a balance skill of greater difficulty would perclude direct comparisons of performance between groups since non-experts would be incapable to execute the skills. The surface of COP oscillations, COP oscillations on the medial-lateral axis, and the length-by-surface confirmed the absence of group difference in physical performance. However, oscillations in the antero-posterior axis during the physical performance of the task were reduced in experts. The arabesque performance involved leaning the body forward, hence the antero-posterior axis corresponds to the main direction of the postural change required to perform the task. The difference in antero-posterior sway oscillations is thus congruent with the group difference in expertise. Reduced sway in experts attests to a greater capacity to maintain their COP within a restricted surface encompassing the orthogonal projection of the participant’s center of gravity. Past research underlined that expert athletes outperformed non-experts across a wide range of balance tasks such as postural stabilization after experimentally-induced perturbations (Gautier et al., 2008a,b,^2009^). Experts are also more prone to switch from visual to proprioceptive balance control strategies and exhibit a more efficient management of the attentional demands associated with the task (Vuillerme et al., 2001; Vuillerme and Nougier, 2004). Postural expertise was also associated with the capacity to recruit secondary proprioceptive information (e.g., otolithic) to assist balance performance when the capacity to engage central processing of primary relevant proprioceptive information was impaired (Bringoux et al., 2000). Present results are in line with previous findings that experts use different postural control strategies than novices, presumably due to a more efficient use of proprioceptive information (Vuillerme et al., 2001; Gautier et al., 2008a,b).

Non-experts exhibited higher VMIQ-2 scores for external visual imagery, which is indicative of a greater difficulty to achieve vivid images in this sensory modality. We found no between-group differences for internal visual imagery and kinesthetic modalities from this questionnaire. As the VMIQ-2 involves general items, why experts outperformed non-experts only for the external visual modality is unclear but could be associated with greater exposure to motor cognition strategies involving an external focus, e.g., observation of performance models. Vividness self-report revealed higher VMI vividness in experts. By contrast, there was no difference between the two groups for kinesthetic MI. Maintaining the arabesque position for long periods of time is not something gymnasts are specifically trained for. Balance is trained dynamically, the arabesque position being mostly encountered during transitions between jumps during floor exercise events. This could explain higher vividness self-reports in experts, who may recognize the task but have limited experience of this movement in the first person. This is in keeping with data from Hardy and Callow (1999) who observed that external visual MI outperformed kinesthetic MI for learning motor skills where form was important compared to motor skills where form was not directly considered to assess performance. There is a well-established relationship between MI ability and the level of motor expertise (Debarnot et al., 2014). Functional brain imaging experiments provided strong evidence that motor expertise was associated with increased recruitment of brain motor networks while performing MI. Visual MI vividness, specifically, was associated with greater precentral and parietal motor activations (Lorey et al., 2011). The present results, therefore, corroborate the scientific literature attesting that motor skills automated as a result of experience-based plasticity yield greater vividness during their mental rehearsal (Kraeutner et al., 2018; Orlandi et al., 2020). Interestingly, the stabilometric correlates of visual MI, but not kinesthetic MI, predicted the surface, MP-length, and AP-length of COP oscillations during the physical performance of the arabesque variation. Vividness self-reports during kinesthetic MI, but not visual MI, predicted the surface and length-by surface of COP oscillation during the actual performance of the balance skill. It is suggested that visual MI enables greater degrees of the embodiment of the postural components of the present gymnastic skill than kinesthetic MI irrespective of the expertise level, whereas vividness achieved during MI under the kinesthetic modality was more reliable to predict the surface and length-by-surface of COP oscillations, both variables indexing of the management of energy expenditure during balance skills. This contradicts findings by Stins et al. (2015), who reported that visual MI elicited reduced postural responses compared to kinesthetic MI and inferred superior embodied cognition under the latter MI modality. In their design, the authors administered non-sporting motor skills, which did not engage the whole body into acrobatic postures. Also, there was no physical practice condition in the design to establish a link with the postural responses under mental rehearsal conditions. These methodological differences might account for such discrepancies. The relationships found between visual MI and postural performance were present at the whole-group level of our sample of participants, i.e., irrespective of participants’ expertise. This result argues against the hypothesis of an influence of the expertise level on the capacity to reproduce the stabilometric correlates of balance skills during MI. This interpretation should be nuanced, however, based on the positive relationship between the years of training practice and the magnitude of postural responses during kinesthetic MI found in experts. Overall, the present results corroborate the hypothesis of a functional relationship between MI and physical practice for balance skills (Collet and Guillot, 2009; Boulton and Mitra, 2015).

Although a link between the stabilometric correlates of MI and actual practice of the balance skill was present in both groups, several differences between expert and non-experts emerged when comparing the stabilometric correlates of MI. While both groups exhibited a similar profile across experimental conditions for the length of COP oscillations in the medial-lateral axes and surface of COP oscillations, this was not the case for the length in the anterior-posterior axis and the length-by-surface. For both stabilometric indexes, there was a decrease as experts engaged in visual or kinesthetic MI compared to the control condition. However, no such changes were present in non-experts: the stabilometric correlates of kinesthetic MI and visual MI were comparable to the control condition. Reduced postural sway in experts may account for both increased attentional focus and more efficient management of energy expenditure (Vuillerme et al., 2001; Vuillerme and Nougier, 2004). Several data attest that the attentional demand required to achieve efficient balance control decreases along with experience (Vuillerme et al., 2001; Vuillerme and Nougier, 2004). Dual-task paradigms evaluating the influence of cognitive tasks on postural control, e.g., verbal responses, attentional focus on external visual cues, memory tasks (Dault et al., 2003; Ehrenfried et al., 2003; Riley et al., 2003), revealed that cognitive operations yield to reallocation of attentional resources from the postural control. Postural control thus becomes controlled by epigenetic postural programs from the brainstem and cerebellum, hence yielding reduced postural sway (see Hamel and Lajoie, 2005 for an illustration in the elderly). The reduced antero-posterior sway and length-by-surface during visual and kinesthetic MI in experts may thus be interpreted as a marker of the motor expertise yielding to the more efficient management of attentional resources during the performance of balance skills.

During physical practice, reduced length of COP oscillations was observed in experts in the anterior-posterior axis. Normalizing stabilometric data recorded during physical practice of the arabesque variation against a stance baseline may be considered a limitation since this is the only condition performed from one leg. While we could have analyzed the stabilometric values without normalization, normalizing against the stance controlled for any individual variations in baseline postural control that could transfer to performance of balance skills. This also enabled the application of homogenous methods across all experimental conditions of the design and should facilitate comparisons in replication studies. Experts achieved, during both visual and kinesthetic MI, distinct postural response patterns from those found non-experts, which may be interpreted as a reflection of their expertise on balance skills. However, high variability in this group under the control condition precludes firm conclusions with regards to a distinct response pattern compared to non-experts. Furthermore, since both MI modalities yielded a comparable decrease in postural sway compared to the control condition, data did not corroborate the working hypothesis of a more embodied nature of kinesthetic MI compared to visual MI. However, the relationship between the number of years of training practice and the stabilometric responses during kinesthetic MI, but not during visual MI, suggests the opposite. Possibly, we faced sample size limitations to achieve a more congruent results pattern. The present experiment brings new insights into the capacity to engage the central nervous system in programming postural commands during MI. Correlation analyses revealed predictive relationships between the stabilometric correlates of visual MI and physical practice of a balance skill, irrespective of the expertise level. This argues in support of an incomplete inhibition of postural motor commands during MI and suggests that experts and non-experts exhibited comparable amounts of postural commands leakage during visual MI. The present findings corroborate the early hypothesis by Boulton and Mitra (2015) that postural commands built during MI may bypass cortical inhibitory processes—although it remains at this point difficult to discriminate whether such postural correlates originate from the incomplete inhibition or facilitation of efferent pathways during MI.

## Conclusion

Conceptual frameworks support that MI and physical practice belong to a continuum extending from the pure mental evocation of the action to its overt execution. According to Stephan and Frackowiak (1996, p. 373), “(…) imagined movements can be viewed as a special form of motor behaviour”. MI thus represents an embodied form of motor cognition (Jeannerod, 2001), with central and peripheral neurophysiological correlates reproducing those recorded during the corresponding motor performance. Contrary to the voluntary motor command signals, anticipatory postural adjustments are largely mediated by subcortical processes. Nonetheless, there is also evidence pointing at a cortical contribution from associative parietal structures (Herold et al., 2017; Di Rienzo et al., 2019). The built-up of anticipatory postural adjustments during motor preparation results in the production of motor commands targeting somatic effectors responsible for balance control. Present data add to the body of evidence supporting the involvement of postural output during MI, presumably anticipatory postural adjustments that are part of motor preparation. Longitudinal research should now disentangle whether adjusting MI interventions according to the expertise level, for instance through the use of distinct modalities, might potentiate the related benefits of MI interventions on postural performances. This issue is of specific relevance in aging populations where declines in cognitive abilities are a corollary of impaired postural control (Clark et al., 1993). This is of specific relevance with regards to the potential efficacy of mental training interventions aiming at preventing falls in the elderly (Segev-Jacubovski et al., 2011).

## Data Availability Statement

The original contributions presented in the study are included in the article/Supplementary Materials, further inquiries can be directed to the corresponding author.

## Ethics Statement

The studies involving human participants were reviewed and approved by Centre de Recherche et d’Innovation sur le Sport. Written informed consent to participate in this study was provided by the participants’ legal guardian/next of kin.

## Author Contributions

AG, FDR, and CC conducted the conceptualization, methodology, supervision, and provided the resources for the design. FDR was involved in data curation and performed the formal analysis. FDR wrote the original draft. Investigations and data curation was performed by QM, FM, TFDK, and PJ. AG and CC reviewed and edited the manuscript. All authors contributed to the article and approved the submitted version.

## Conflict of Interest

The authors declare that the research was conducted in the absence of any commercial or financial relationships that could be construed as a potential conflict of interest.

## Publisher’s Note

All claims expressed in this article are solely those of the authors and do not necessarily represent those of their affiliated organizations, or those of the publisher, the editors and the reviewers. Any product that may be evaluated in this article, or claim that may be made by its manufacturer, is not guaranteed or endorsed by the publisher.

## Footnotes

^1^ https://www.wma.net/policies-post/wma-declaration-of-helsinki-ethical-principles-for-medical-research-involving-human-subjects/

^2^ https://cran.r-projects.org/web/packages/emmeans/index.html
